# WP1130 Enhances TRAIL-Induced Apoptosis through USP9X-Dependent miR-708-Mediated Downregulation of c-FLIP

**DOI:** 10.3390/cancers11030344

**Published:** 2019-03-11

**Authors:** Seok Kim, Seon Min Woo, Kyoung-jin Min, Seung Un Seo, Tae-Jin Lee, Peter Kubatka, Dong Eun Kim, Taeg Kyu Kwon

**Affiliations:** 1 Department of Immunology, School of Medicine, Keimyung University, 1095 Dalgubeoldaero, Dalseo-Gu, Daegu 42601, Korea; kimseok216@naver.com (S.K.); woosm724@gmail.com (S.M.W.); kyoungjin.min@gmail.com (K.-j.M.); ssu3885@gmail.com (S.U.S.); 2 Department of Anatomy, College of Medicine, Yeungnam University, Daegu 42415, Korea; tjlee@med.yu.ac.kr; 3 Department of Medical Biology, Jessenius Faculty of Medicine, Comenius University in Bratislava, Martin 03601, Slovakia; kubatkap@gmail.com; 4 Department of Experimental Carcinogenesis, Division of Oncology, Biomedical Center Martin, Jessenius Faculty of Medicine, Comenius University in Bratislava, Martin 03601, Slovakia; 5 Department of Otolaryngology, School of Medicine, Keimyung University, 1095 Dalgubeoldaero, Dalseo-Gu, Daegu 42601, Korea; entkde@dsmc.or.kr

**Keywords:** WP1130, TRAIL, c-FLIP, DR5, MicroRNA

## Abstract

WP1130, a partially selective deubiquitinases (DUB) inhibitor, inhibits the deubiquitinating activities of USP5, USP9X, USP14, USP37, and UCHL1. In this study, we investigate whether WP1130 exerts sensitizing effect on TNF-related apoptosis-inducing ligand (TRAIL)-induced apoptosis in human renal carcinoma cells. Combinations of WP1130 and TRAIL significantly induced apoptosis in renal carcinoma, lung carcinoma and hepatocellular carcinoma cells, but not in normal cells (human mesangial cells (MC) and normal mouse kidney cells (TCMK-1)). The downregulation of c-FLIP protein expression was involved in combined treatment-induced apoptosis. WP1130-induced c-FLIP downregulation was regulated by microRNA (miR)-708 upregulation via inhibition of USP9X. Interestingly, knockdown of USP9X markedly induced c-FLIP downregulation, upregulation of miR-708 expression and sensitivity to TRAIL. Furthermore, ectopic expression of USP9X prevented c-FLIP downregulation and apoptosis upon combined treatment. In sum, WP1130 sensitized TRAIL-induced apoptosis through miR-708-mediated downregulation of c-FLIP by inhibition of USP9X.

## 1. Introduction

The ubiquitin proteasome system (UPS) is a major regulator for the targeted degradation of proteins [1,2]. UPS is highly dynamic modulated by ubiquitination and deubiquitination. Deubiquitinases (DUBs) consist of five cysteine proteases (ubiquitin-specific proteases (USPs), ubiquitin C-terminal hydrolases (UCHs), ovarian tumor proteases (OTUs), Machado-Joseph domain proteases (MJD), and MIU-containing novel DUB family (MINDY) protease), one zinc metallo-iso-peptidase and the herpes virus tegument USPs (e.g., JAB1/MPN/MOV34 metalloproteases/JAMM) [3,4,5]. DUBs represent an attractive potential target for the development of cancer therapies due to their important roles in multiple cellular processes such as protein stability, localization and cellular homeostasis. WP1130 acts as a partially selective DUB inhibitor that decreases the activity of USP5, USP9X, USP14, USP37, and UCHL1 [6]. The DUB inhibitory effect of WP1130 has been considered as a potential sensitizer to overcome chemoresistance. WP1130 decreases Mcl-1 expression, resulted in enhancement of radiation-induced apoptosis in non-small cell lung cancer cells [7] and overcomes resistance against cisplatin or doxorubicin via downregulation of p53 or Mcl-1 expression in cancer cells [8,9,10]. The anti-cancer effects of WP1130 rely primarily on the inhibition of USP9X, and inhibition of USP9X by WP1130 induces downregulation of Mcl-1 and XIAP expression [7,11,12,13]. However, the anti-cancer drug sensitizing mechanism of WP1130 is unclear.

Tumor necrosis factor-related apoptosis-inducing ligand (TRAIL), one of the TNF ligand family members, selectively induces apoptosis by binding to death receptors (DR4 and DR5) in cancer cells [14,15]. However, TRAIL treatment has little or insignificant effects on several types of cancers through a variety of mechanisms [16]. Resistance of TRAIL is mainly due to following factors: downregulation of DRs expression, upregulation of decoy receptors (DcR1 and DcR2) and anti-apoptotic proteins, and activation of survival signal pathways [17,18,19,20]. To overcome these limitations, several studies have shown that targeted delivery of TRAIL and combined treatment with chemotherapeutic drugs can overcome TRAIL resistance [21]. Therefore, discovery of novel sensitizers for TRAIL are urgently required to overcome TRAIL resistance.

In this study, we show the molecular mechanisms and practical role of WP1130 as a TRAIL sensitizer. The inhibition of USP9X by WP1130 sensitizes Caki cells to TRAIL-mediated apoptosis through miR-708-dependent c-FLIP downregulation at posttranscriptional level. 

## 2. Results

### 2.1. WP1130 Sensitizes Caki Cells to TRAIL-Induced Apoptosis through Caspase Activation

**Figure 1:**
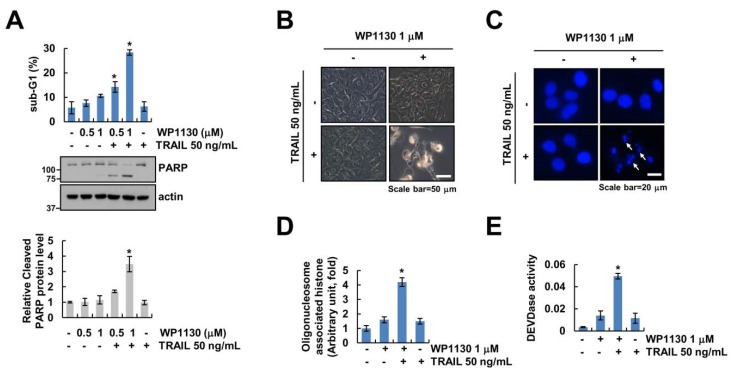

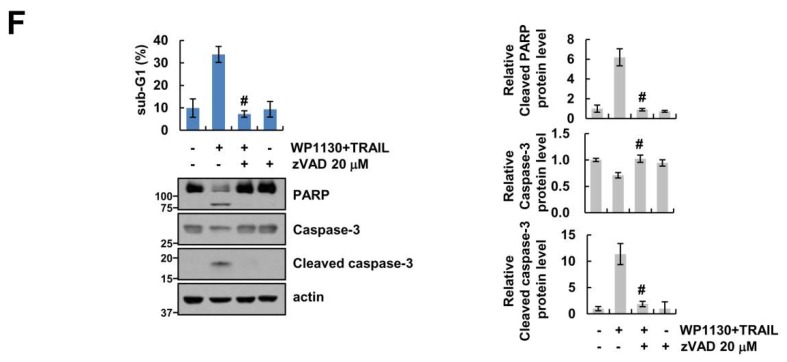
Combined treatment of the deubiquitinase inhibitor WP1130 with TRAIL induces apoptosis in Caki cells. (**A**) Caki cells were treated with WP1130 (0.5, 1 μM) and/or 50 ng/mL TRAIL for 24 h. (**B**–**E**) The cell morphology (**B**), 4’, 6’-diamidino-2-phenylindole image (**C**), DNA fragmentation (**D**), and DEVDase (caspase-3) activity (**E**) were examined using microscopy or assay kits in Caki cells treated with 50 ng/mL TRAIL in the presence or absence of 1 μM WP1130 for 24 h. (**F**) Caki cells were treated with 1 μM WP1130 plus 50 ng/mL TRAIL for 24 h in the presence or absence of pretreatment with z-VAD-fmk (zVAD) for 30 min. Apoptosis and protein levels were determined by flow cytometry and western blotting. The values in graphs (**A**,**D**–**F**) represent the mean ± SD from three independent experiments. * *p* < 0.01 compared with the control. # *p* < 0.01 compared with the WP1130 plus TRAIL.

We investigated whether WP1130, which inhibits DUBs activity, exerts synergistic effects on TRAIL-induced apoptosis in renal carcinoma Caki cells. WP1130 alone (0.5, 1 μM) and TRAIL alone (50 ng/mL) did not increase apoptosis (Figure 1A). However, combination of WP1130 plus TRAIL significantly increased cleavage of poly (ADP-ribose) polymerase (PARP) and accumulation of the sub-G1 population (Figure 1A). Therefore, 1 μM WP1130 was used for further combined treatment with 50 ng/mL TRAIL. Although single treatment did not yield apoptotic events, combined treatment caused typical apoptotic morphologies, nuclear condensation and DNA fragmentation (Figure 1B–D). 

Moreover, combination of WP1130 and TRAIL treatment significantly induced caspase-3 (DEVDase) activation (Figure 1E). To confirm whether caspase activation is associated with induction of apoptotic cell death by WP1130 plus TRAIL, we used the pan-caspase inhibitor (z-VAD-fmk). z-VAD significantly inhibited combination of WP1130 and TRAIL-induced apoptosis and cleavage of PARP (Figure 1F). Therefore, these data suggest that combined WP1130 and TRAIL treatment stimulates apoptotic cell death in caspase-dependent manner in Caki cells.

### 2.2. Effect of WP1130 on Regulation of Apoptosis-Related Proteins

**Figure 2:**
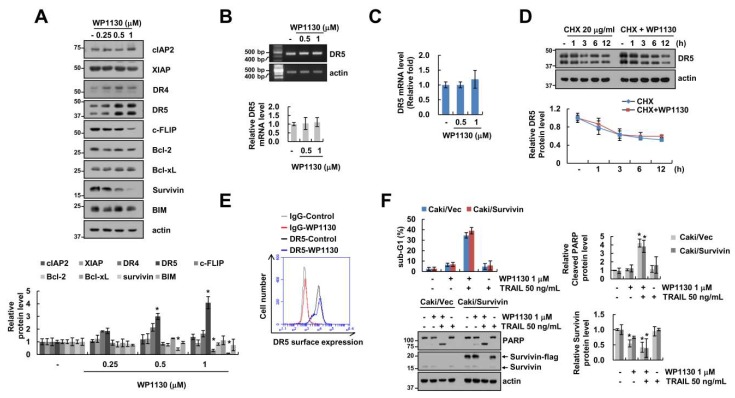
Effect of WP1130 on apoptosis-related proteins in Caki cells. (**A**) The dose-dependent effect of WP1130 (0.25–1 μM) treatment for 24 h on the protein expression levels of cIAP2, XIAP, DR4, DR5, c-FLIP, Bcl-2, Bcl-xL, survivin, and BIM in Caki cells as demonstrated using western blotting. (**B**,**C**) The dose-dependent effect of WP1130 (0.5–1 μM) treatment for 24 h on DR5 mRNA levels in Caki cells using reverse transcription polymerase chain reaction (RT-PCR, **B**) and quantitative PCR (qPCR, **C**), respectively. (**D**) Caki cells were treated with or without 1 μM WP1130 in the presence of 20 μg/mL cycloheximide (CHX) for the indicated time periods. (**E**) Cell surface DR5 expression levels were measured by flow cytometry analysis in Caki cells after treatment with 1 μM WP1130 for 24 h. (**F**) Vector cells (Caki/Vec) and survivin-overexpressing cells (Caki/Survivin) were treated with 1 μM WP1130, 50 ng/mL TRAIL, and a combination of both for 24 h. Apoptosis and protein levels were determined by flow cytometry and western blotting, respectively. The values in graph (**A**–**D**,**F**) represent the mean ± SD from three independent experiments. * *p* < 0.01 compared with the control.

To investigate the basic mechanisms involving WP1130-mediated TRAIL sensitization, we analyzed the change in apoptosis-related proteins induced by WP1130. c-FLIP and survivin expression levels decreased after 24 h of WP1130 treatment, while other proteins (cIAP2, XIAP, Bcl-2, Bcl-xL, Bim and DR4) were not changed by WP1130. Interestingly, WP1130 induced upregulation of DR5 protein expression (Figure 2A), but did not increase DR5 mRNA levels (Figure 2B,C). Because WP1130 did not change DR5 mRNA expression, we investigated the effect of WP1130 on DR5 protein stability. Cells were treated with cycloheximide (CHX), a protein biosynthesis inhibitor, followed by addition of WP1130. CHX and WP1130 in combination revealed a similar degradation pattern compared with CHX alone (Figure 2D). Increased DR5 expression on the cellular surface is a critical factor in the induction of DR-mediated apoptosis. Unexpectedly, WP1130 did not increase DR5 surface expression (Figure 2E). These data suggest that DR5 upregulation by WP1130 in the cytosol might not be critical for WP1130-mediated TRAIL sensitization. Next, to explore the role of survivin downregulation in the induction of apoptosis by WP1130 plus TRAIL treatments, we employed survivin-overexpressing cells. However, combination of WP1130 and TRAIL still increased apoptosis in survivin-overexpressing cells (Figure 2F). These data indicate that downregulation of survivin by WP1130 is not associated with TRAIL sensitization. 

### 2.3. Downregulation of c-FLIP by WP1130 is Involved in TRAIL-Induced Apoptosis

**Figure 3:**
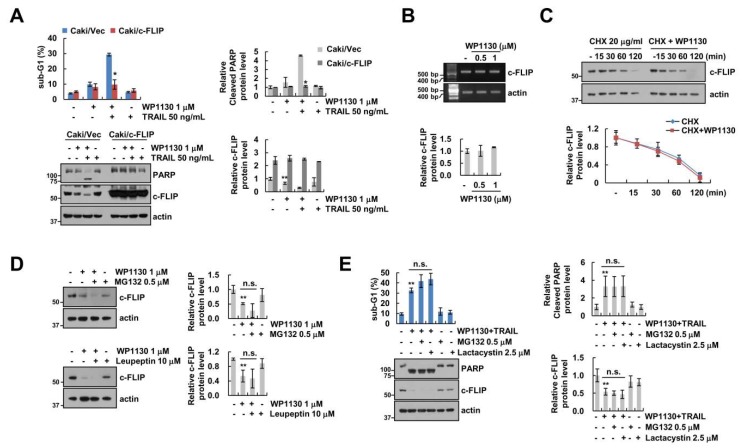
Downregulation of c-FLIP is involved in WP1130 plus TRAIL-induced apoptosis. (**A**) Vector cells (Caki/Vec) and c-FLIP-overexpressing cells (Caki/c-FLIP) were treated with 1 μM WP1130, 50 ng/mL TRAIL, and combinations of both for 24 h. (**B**) The dose-dependent effect of WP1130 (0.5–1 μM) treatment for 24 h on the mRNA levels of c-FLIP in Caki cells as demonstrated using RT-PCR. (**C**) Caki cells were treated with or without 1 μM WP1130 in the presence of 20 μg/mL CHX for the indicated time periods. (**D**) Caki cells were treated with 1 μM WP1130 for 24 h in the presence or absence of pretreatment with 0.5 μM MG132 and 10 μM leupeptin for 30 min. (**E**) Caki cells were treated with 1 μM WP1130 plus 50 ng/mL TRAIL for 24 h in the presence or absence of pretreatment with 0.5 μM MG132 and 2.5 μM lactacystin for 30 min. Apoptosis and protein levels were determined by flow cytometry and western blotting, respectively. The values in the graph (**A**–**E**) represent the mean ± SD from three independent experiments. * *p* < 0.01 compared with the WP1130 plus TRAIL-treated Caki/Vector. ** *p* < 0.05 compared with the control. n.s. = no significant.

Next, we investigated the effect of c-FLIP downregulation on combined treatment-induced apoptosis. Ectopic expression of c-FLIP significantly prevented combination of WP1130 and TRAIL-induced apoptosis and PARP cleavage (Figure 3A). These data suggest that c-FLIP reduction is essential for combination of WP1130 and TRAIL-induced apoptosis. Interestingly, WP1130 did not alter levels of c-FLIP mRNA expression (Figure 3B). Therefore, we analyzed the downregulation of c-FLIP by WP1130 through protein stability. WP1130 plus CHX showed a similar degradation pattern to as CHX treatment alone (Figure 3C). WP1130 did not enhance the c-FLIP degradation rate. To further examine the proteasomal or lysosomal involvement in WP1130-mediated c-FLIP degradation, we used a proteasome inhibitor (MG132) and lysosomal inhibitor (leupeptin). However, WP1130-induced c-FLIP downregulation was not abolished by both inhibitors (Figure 3D). In addition, pretreatment with proteasome inhibitors did not inhibit combined WP1130 and TRAIL treatment-induced apoptosis (Figure 3E). These data indicate that WP1130 did not regulate c-FLIP expression via transcriptional and ubiquitin-proteasome activities. 

### 2.4. WP1130 Increases miR-708-Mediated c-FLIP Downregulation

**Figure 4:**
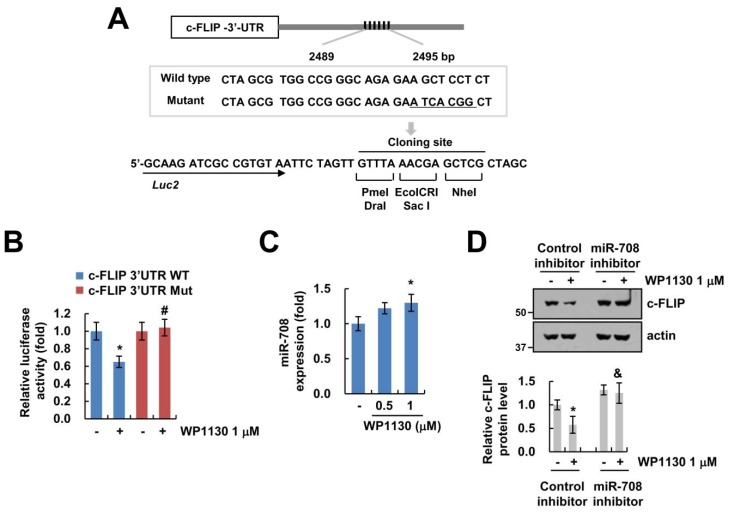
WP1130 decreases c-FLIP expression by increasing miR-708. (**A**) The conserved binding sites of miR-708 are indicated in the c-FLIP 3’UTR and the mutated nucleotides in c-FLIP 3’UTR mutant. (**B**) Luciferase reporter gene for c-FLIP 3’UTR wild-type (WT) and mutant (Mut) transfected, and cells were further cultured for 24 h. Cells were treated with 1 μM WP1130 for 24 h, and luciferase activity was analyzed. (**C**) The dose-dependent effect of WP1130 (0.5–1 μM) treatment for 24 h on the miR-708 expression levels in Caki cells as demonstrated using qPCR. (**D**) miRNA inhibitor, negative control (control inhibitor) and miR-708 inhibitor were transfected and then further culture for 24 h. Cells were treated with 1 μM WP1130 for 24 h. Protein levels were determined by Western blotting. The values in graph (**B**–**D**) represent the mean ± SD from three independent experiments. * *p* < 0.01 compared with the control. # *p* < 0.01 compared with the WP1130-treated c-FLIP 3’UTR WT. & *p* < 0.01 compared with the WP1130-treated control inhibitor.

We previously reported that miR-708 binds to the 3’-untranslated region (3’UTR), reducing c-FLIP expression, and nucleotides 2489 to 2495 in c-FLIP 3’UTR plays a critical role in miR-708 binding (Figure 4A) [22,23]. Therefore, we used luciferase constructs with wild type and mutant miR-708 binding sites in c-FLIP 3’UTR (Figure 4A). WP1130 markedly suppressed the luciferase activity of c-FLIP 3’UTR wild type, but not c-FLIP 3’UTR mutant (Figure 4B). In addition, WP1130 increased miR-708 expression levels (Figure 4C). To verify that the increase in miR-708 mediated by WP1130 involves c-FLIP downregulation, we used a miR-708 inhibitor. The miR-708 inhibitor reversed WP1130-induced c-FLIP downregulation (Figure 4D). Thus, these data indicate that upregulation of miR-708 by WP1130 induced downregulation of c-FLIP expression. 

### 2.5. USP9X is a Major Target of miR-708-Mediated c-FLIP Downregulation and Induction of TRAIL Sensitization by WP1130

**Figure 5:**
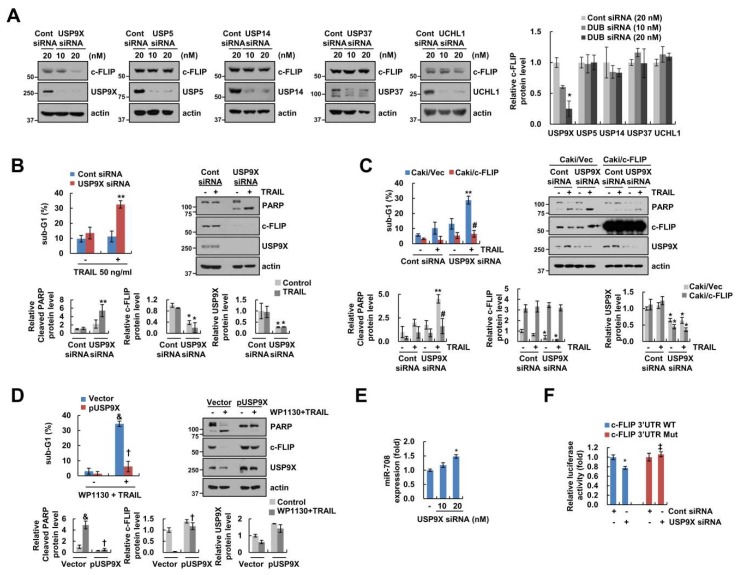
Knockdown of USP9X increases TRAIL sensitivity through miR-708-dependent downregulation of c-FLIP expression. (**A**) siRNAs against Control (Cont), USP9X, USP5, USP14, USP37, and UCHL1 transfected into Caki cells for 24 h. (**B**) Cont siRNA or USP9X siRNA transfected into Caki cells, and then cells were treated with 50 ng/mL TRAIL for 24 h. (**C**) Caki siRNA or USP9X siRNA transfected into Caki/Vec and Caki/c-FLIP cells, and then cells were treated with 50 ng/mL TRAIL for 24 h. (**D**) The constructs for pDESTS1 (Vector) or pDESTS1-mUSP9X (pUSP9X) were transfected, and then cells were treated with 1 μM WP1130 plus 50 ng/mL TRAIL for 24 h. (**E**) Cont siRNA or USP9X siRNA transfected into Caki cells, and miR-708 expression levels were determined by qPCR. (**F**) The luciferase reporter gene for c-FLIP 3’UTR WT or Mut cotransfected with or without Cont siRNA and USP9X siRNA into Caki cells, and luciferase activity was analyzed. Apoptosis and protein levels were determined by flow cytometry and western blotting. The values in graph (**A**–**F**) represent the mean ± SD from three independent experiments. * *p* < 0.01 compared with control siRNA. ** *p* < 0.01 compared with TRAIL-treated control siRNA. # *p* < 0.01 compared with TRAIL-treated USP9X siRNA in Caki/Vec. & *p* < 0.05 compared with control in Caki/Vec. † *p* < 0.01 compared with WP1130 plus TRAIL-treated Caki/Vec. ‡ *p* < 0.01 compared with the USP9X siRNA-treated c-FLIP 3’UTR WT. Black arrow (**C**) indicated non-specific band.

WP1130 decreases DUB activity of USP5, USP9X, USP14, USP37, and UCHL1. To further assess which DUB was involved in downregulation of c-FLIP by WP1130, we used specific siRNA for each of the above DUBs. As shown in Figure 5A, knockdown of USP9X decreased c-FLIP protein levels, whereas knockdown of USP5, USP14, USP37 or UCHL1 did not alter c-FLIP protein levels (Figure 5A). Next, we investigated the importance of USP9X in the sensitizing effect of WP1130 plus TRAIL-induced apoptosis. Inhibition of USP9X by siRNA sensitized Caki cells to TRAIL-mediated apoptosis through downregulation of c-FLIP expression (Figure 5B). Next, we investigated the effect of c-FLIP downregulation on TRAIL sensitization by USP9X inhibition. Ectopic expression of c-FLIP markedly prevented TRAIL-mediated apoptosis by USP9X inhibition (Figure 5C). In addition, USP9X overexpression markedly prevented c-FLIP downregulation, PARP cleavage and the generation of sub-G1 population induced upon WP1130 plus TRAIL combination treatment (Figure 5D). To further examine the involvement of USP9X inhibition in miR-708-mediated c-FLIP downregulation by WP1130, we analyzed miR-708 expression and c-FLIP 3’-UTR luciferase activity. Knockdown of USP9X increased miR-708 expression and decreased wild type c-FLIP 3’-UTR luciferase activity (Figure 5E,F). In contrast, luciferase activity of the 3’-UTR mutant in c-FLIP was not changed by USP9X inhibition (Figure 5F). Therefore, these results suggest that inhibition of USP9X by WP1130 is involved in miR-708-mediated downregulation of c-FLIP, resulting in increased TRAIL sensitivity.

### 2.6. Effect of Combinations of WP1130 and TRAIL on Apoptosis in Various Cell Lines

**Figure 6:**
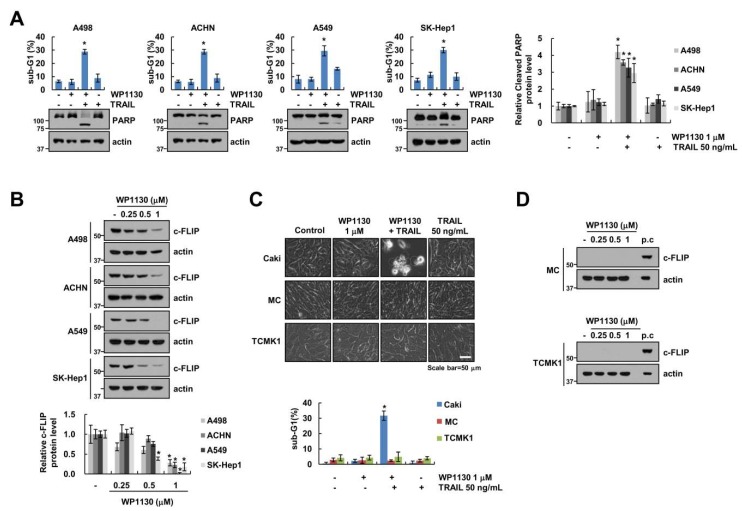
The effect of WP1130 and TRAIL on apoptosis in other carcinoma cells and normal cells. (**A**) A498, ACHN, A549 and SK-Hep1 cells were treated with 1 μM WP1130, 50 ng/mL TRAIL, and combinations of both for 24 h. (**B**) The dose-dependent effect of WP1130 (0.25–1 μM) treatment for 24 h on the c-FLIP protein expression levels in A498, ACHN, A549 and SK-Hep1 cells. (**C**) Cell morphology and apoptosis levels were determined by interference light microscopy and flow cytometry. (**D**) The dose-dependent effect of WP1130 (0.25–1 μM) treatment for 24 h on protein expression levels in MC and TCMK1 cells as demonstrated (positive control (p.c); no treatment in Caki cells). Apoptosis and protein levels were determined by flow cytometry and Western blotting. The values in graph (**A**–**C**) represent the mean ± SD from three independent experiments. * *p* < 0.01 compared with control.

To generalize these events, we examined the effect of WP1130 on TRAIL-induced apoptosis in other carcinoma cells (renal carcinoma; ACHN and A498, lung carcinoma; A549, and hepatocellular carcinoma; SK-Hep1). In all cell types, WP1130 and TRAIL combinations increased apoptosis and PARP cleavage (Figure 6A). Moreover, WP1130 induced the reduction of c-FLIP protein levels in all tested cells (Figure 6B). In contrast, combination WP1130 and TRAIL treatment did not induce apoptotic morphology and a sub-G1 population in normal human mesangial cells (MC) and normal mouse kidney cells (TCMK1) (Figure 6C). In addition, we did not detect c-FLIP protein levels in normal cells (Figure 6D). These data indicate that WP1130 selectively sensitizes cancer cells to TRAIL-mediated apoptosis.

## 3. Discussion

Our results demonstrated that WP1130 and TRAIL in combination induced apoptosis via downregulation of c-FLIP in cancer cells. WP1130 increased miR-708 expression followed by reduction of c-FLIP expression. We found that inhibition of USP9X DUB activity by WP1130 was associated with TRAIL sensitization via downregulation of c-FLIP expression. Therefore, these results suggest that WP1130 could be an attractive TRAIL sensitizer. 

The role of the WP1130 involves reducing the expression of anti-apoptotic Bcl-2 proteins and IAP family (XIAP and survivin) [7,11,13]. In our study, WP1130 also decrease survivin expression (Figure 2A). However, downregulation of survivin by WP1130 was not involved in WP1130 combined with TRAIL-induced apoptosis (Figure 2F). Furthermore, WP1130 did not alter Bcl-2, Bcl-xL and XIAP (Figure 2A). Interestingly, WP1130 downregulated c-FLIP expression in Caki cells (Figure 2A), and reduced c-FLIP expression was involved in WP1130-mediated TRAIL sensitization (Figure 3A). c-FLIP expression is transcriptionally or posttranslationally regulated. Multiple transcriptional factors including NF-κB [24], AP-1 [25], Sp1 [26], p53 [27] and FOXO3a [28] modulate c-FLIP mRNA expression, and ubiquitin E3 ligases (Itch and Cbl) eventually increase degradation of c-FLIP through activation of the ubiquitin-proteasome pathway [29,30]. Recently, Hsu et al. reported that deltex1 E3 ligase degrades c-FLIP protein levels, resulting in the increase in Fas ligand- and TRAIL-mediated apoptosis in gastric cancer cells [31]. However, WP1130-mediated c-FLIP downregulation was not regulated at the transcriptional or posttranslational levels (Figure 3B,C). Furthermore, proteasome and lysosome inhibitors did not reverse c-FLIP degradation by WP1130 treatment (Figure 3D). In addition, c-FLIP protein stability was not altered by WP1130 treatment (Figure 3C). These findings suggest that WP1130-induced c-FLIP downregulation is not associated with transcriptional regulation or the ubiquitin-proteasome pathway. MicroRNAs (miRNA) are small noncoding RNAs that negatively regulate the expression of multiple genes by translational repression [32]. Several studies have sought to identify the miRNA targeting c-FILP mRNA, such as miR-1246, miR-320a, miR-196b-5p and miR-512-3p [33,34]. Recently, our group also reported that miR-708 binds to 3’-UTR in c-FLIP and inhibited c-FLIP expression followed by the increased TRAIL-induced apoptosis [22,23]. WP1130 also induced upregulation of miR-708 expression (Figure 4C), and the miR-708 inhibitor reversed WP1130-induced c-FLIP downregulation (Figure 4D). Intratumoral delivery of miR-708 leads to repress tumorigenicity in a renal cancer xenograft model [35]. These data suggest that miR-708 plays a critical role as a pro-apoptotic miRNA in renal cancers. However, further studies are required to elucidate the molecular mechanism for WP1130-induced miR-708 expression.

WP1130 acts as a cell-permeable DUB inhibitor and contains anti-proliferative and apoptotic effects against various tumor cells [6,36,37]. Recently, several reports demonstrated that inhibition of USP9X and/or USP5 by WP1130 leads to apoptotic cell death in malignant peripheral nerve sheath tumors and multiple myeloma [38,39]. Moreover, WP1130 increases sensitivity to various chemotherapeutic agents such as cisplatin, doxorubicin and gemcitabine through inhibition of USP9X activity [8,9,10,40]. Our data suggested that WP1130 suppressed c-FLIP expression via inhibition of USP9X DUB activity. Inhibition of only USP9X, but not the other DUBs (USP5, USP14, UPS37 and UCHL1), by RNA interference induced downregulation of c-FLIP expression (Figure 5A). In addition, knockdown of USP9X increased miR-708 expression and inhibited luciferase activity of wild type 3’-UTR in c-FLIP (Figure 5E,F). Furthermore, overexpression of USP9X prevented WP1130 plus TRAIL-induced apoptosis and c-FLIP downregulation (Figure 5D). Therefore, these findings suggest that WP1130 sensitized Caki cells to TRAIL-mediated apoptosis through miR-708-mediated down-regulation of c-FLIP by inhibition of USP9X.

## 4. Materials and Methods

### 4.1. Cell Cultures and Materials

Human renal carcinoma cells (Caki, ACHN and A498), human lung cancer cells (A549), and human hepatocellular carcinoma cells (SK-Hep1) were obtained from the American Type Culture Collection (Manassas, VA, USA). Cells were cultured in Dulbecco’s modified Eagle’s medium (DMEM) or Roswell Park Memorial Institute (RPMI) 1640 (Welgene, Gyeongsan, Korea) containing 10% fetal bovine serum, 5% penicillin/streptomycin (Welgene) and 100 μg/mL gentamicin (Thermo Fisher Scientific, Waltham, MA, USA). WP1130 was purchased from Cayman Chemical (Ann Arbor, MI, USA). z-VAD-fmk, human recombinant TRAIL and anti-survivin antibody were obtained from R&D System (Minneapolis, MN, USA). Cell Signaling Technology (Beverly, MA, USA) supplied anti-PARP, anti-cleaved caspase-3, anti-DR5, anti-Bcl-xL, anti-USP14, and anti-UCHL1 antibodies. BD Biosciences (San Jose, CA, USA) provided anti-caspase-3, anti-Bim, and anti-XIAP antibodies. Santa Cruz Biotechnology (Santa Cruz, CA, USA) provided anti-cIAP2 and anti-USP14 antibodies. Enzo Life Sciences (San Diego, CA, USA) supplied the anti-c-FLIP antibody. Abcam (Cambridge, MA, USA) supplied the anti-DR4, anti-Bcl-xL, and anti-USP5 antibodies and Abnova (Taipei City, Taiwan) provided the anti-USP9X antibody. MG132 was purchased from Calbiochem (San Diego, CA, USA), and lactacystin was obtained from Biomol Research Laboratories (Plymouth Meeting, PA, USA). Cycloheximide, leupeptin, and anti-actin antibody were obtained from Sigma Chemical Co. (St. Louis, MO, USA). mirVanaTM miRNA inhibitor, negative control (control inhibitor) and mirVana™ miRNA inhibitor (miR-708 inhibitor) was purchased from Invitrogen (Carlsbad, CA, USA).

### 4.2. Western Blot Analysis and Flow Cytometry Analysis

Total lysates were obtained using-modified RIPA lysis buffer as described previously [41,42,43]. The proteins were separated by SDS-PAGE and transferred onto an Immobilon-P membrane (GE Healthcare Life Science, Pittsburgh, PO, USA). The membranes were incubated with 5% nonfat milk for 30 min and then overnight with the primary antibodies. After overnight incubation, the membranes were incubated with secondary antibodies for 2 h, and were exposed using an enhanced chemiluminescence Western blot kit (EMD Millipore, Darmstadt, Germany). All protein band intensity was measured using ImageJ. For apoptosis analysis, cells were fixed with 100% ethanol for 2 h at 4 °C. Next, cells were incubated with RNase for 30 min at 37 °C and stained with propidium iodide. DNA content was measured by flow cytometry (BD Biosciences).

### 4.3. DAPI Staining, DNA Fragmentation Assay and Caspase Activity Assay

For DAPI staining, cells were stained with 300 nM 4’, 6’-diamidino-2-phenylindole solution (Roche, Mannheim, Germany), and we analyzed fluorescence images using fluorescence microscopy (Carl Zeiss, Jena, Germany). We performed the DNA fragmentation assay as described in our previous studies [44]. To evaluate caspase-3 (DEVDase) activity, 20 μg of lysates were incubated with reaction buffer as described in our previous studies [44]. 

### 4.4. Reverse Transcription-Polymerase Chain Reaction (RT-PCR) and Quantitative PCR (qPCR)

To obtain cDNA, total RNA was prepared using the TriZol reagent (Life Technologies, Gaithersburg, MD, USA), and M-MLV reverse transcriptase (Gibco-BRL, Gaithersburg, MD, USA) was used. For PCR, we used DNA Taq polymerase with primers targeting DR5, c-FLIP and actin [45,46]. The amplified products were separated by electrophoresis on a 2% agarose gel and detected under UV light. All mRNA band intensity was measured using ImageJ. For qPCR, SYBR Fast qPCR Mix (Takara Bio Inc., Shiga, Japan) was used, and reactions were performed on Thermal Cycler Dice® Real Time System III (Takara Bio Inc., Shiga, Japan). We used DR5, c-FLIP and actin primers for qPCR: DR5 (sense) 5′-AGA CCC TTG TGC TCG TTG TC-3′ and (antisense) 5′-TTG TTG GGT GAT CAG AGC AG-3′, c-FLIP (sense) 5′-CGC TCA ACA AGA ACC AGT G-3′ and (antisense) 5′-AGG GAA GTG AAG GTG TCT C-3′, and actin (sense) 5′-CTA CAA TGA GCT GCG TGT G-3′ and (antisense) 5′-TGG GGT GTT GAA GGT CTC-3′. We calculated the threshold cycle number (Ct) of each gene using actin as the reference gene, and we reported the delta-delta Ct values of the genes.

### 4.5. Detection of DR5 on Cell Surface

To examine DR5 expression on cell surface, cells were incubated with DR5-phycoerythrin (Abcam) in PBS including 10% FCS and 1% sodium azide. We analyzed the surface expression of DR5 using flow cytometry as described in our previous study [47]. 

### 4.6. Construction of Stable Cell Lines

The Caki cells were transfected in a stable manner with the pcDNA 3.1/survivin-flag plasmid, pcDNA 3.1/c-FLIP plasmid or control plasmid pcDNA 3.1 vector using Lipofectamine^TM^ 2000 (Invitrogen). After 48 h of incubation, transfected cells were selected in cell culture medium containing 700 μg/mL G418 (Invitrogen). After 2 or 3 weeks, single independent clones were randomly isolated, and each individual clone plated separately. After clonal expansion, cells from each independent clone were tested for survivin and c-FLIP expression by western blotting.

### 4.7. Detection of miR-708 Expression

RT reactions were performed using a TaqMan® MicroRNA Reverse Transcription Kit and TaqMan® MicroRNA assays. We analyzed miR-708 expression using the TaqMan Universal PCR Master Mix No AmpErase UNG (Applied Biosystems, Foster City, CA, USA).

### 4.8. Luciferase Assay and DNA Transfection

The c-FLIP 3’-UTR luciferase promoter was kindly provided by Dr. TJ Lee (Yeungnam University, Daegu, Korea). Caki cells were transfected with c-FLIP 3’-UTR luciferase promoter using Lipofectamine^TM^ 2000 (Invitrogen, Carlsbad, CA, USA). After transfection, cells were treated with 1 μM WP1130 for 24 h, and then lysates were incubated with luciferase substrate, luciferin (Promega, Madison, WI, USA). pDESTS1-mUSP9X or pDESTS1 vector plasmids used for our study. pDESTS1 mUSP9X was kindly provided by Dr. Rakesh Srivastava. Lipofectamine^TM^ 2000 was used to transfect the plasmids (Invitrogen).

### 4.9. RNA Interference

Green fluorescent protein (GFP, control) siRNA was purchased from Bioneer (Daejeon, Korea). USP9X, USP5, UCHL1, USP14, and USP37 siRNA was obtained from Santa Cruz Biotechnology. Each siRNA was transfected into cells using Lipofectamine® RNAiMAX Reagent (Invitrogen).

### 4.10. Statistical Analysis

The data were analyzed using a one-way ANOVA and post hoc comparisons (Student-Newman-Keuls) using the Statistical Package for Social Sciences 22.0 software (SPSS Inc., Chicago, IL, USA). 

## 5. Conclusions

Our study provides the evidence that inhibition of USP9X by WP1130 induces miR-708-mediated c-FLIP suppression in cancer cells. Downregulation of c-FLIP expression by WP1130 induces sensitization of TRAIL-induced apoptosis. Therefore, WP1130 may represent an attractive sensitizer in TRAIL-resistant cancer cells.

## Abbreviations

TRAIL	Tumor necrosis factor-related apoptosis-inducing ligand
DR5	death receptor 5
PARP	poly (ADP-ribose) polymerase
c-FLIP	Cellular FADD-like IL-1β-converting enzyme-inhibitory protein
3’-UTR	3’-untranslated region
